# Blocking indolamine 2,3 dioxygenase mediated rebound immune suppression improves systemic anti-tumor effects of radio-immunotherapy

**DOI:** 10.1186/2051-1426-3-S2-P368

**Published:** 2015-11-04

**Authors:** Arta M Monjazeb, Michael Kent, Steven K Grossenbacher, William Murphy

**Affiliations:** 1 UC Davis Comprehensive Cancer Center, Sacramento, CA, USA; 2 UC Davis, Davis, CA, USA; 3 UC Davis, Sacramento, CA, USA

## 

Immunotherapy can paradoxically up-regulate immunosuppressive pathways, a phenomenon we term “rebound immunosuppression”. One such pathway is the immunosuppressive enzyme indolamine-2,3-dioxygenase (IDO). Here, we postulated that IDO blockade will improve immunotherapy efficacy by preventing rebound immune-suppression. It has been previously shown that immunotherapy consisting of intralesional CpG and radiotherapy can induce objective systemic responses but that patients whose tumors induce regulatory Tregs are unlikely to respond. We suggest that IDO induced rebound immune suppression limits the effectiveness of this combination by preventing rebound immune suppression. In tumor-bearing mice, local IDO expression markedly increased after treatment with local radiotherapy + intratumoral CpG or other immunotherapies. The addition of IDO blockade to radiotherapy + CpG decreased IDO expression and activity, reduced tumor growth, and reduced immunosuppressive factors, including Tregs, in the tumor microenvironment but not systemically. This triple combination did induce systemic anti-tumor effects decreasing metastases and improving survival in a CD8+ T cell dependent manner. IDO blockade and/or CPG were ineffective without radiotherapy. We evaluated this novel triple therapy in a canine clinical trial, since spontaneous canine malignancies closely reflect human cancer. Mirroring the results in mice, therapy was well tolerated, reduced local tumor immunosuppression, and induced robust systemic anti-tumor effects. Disease response or stability was observed in 80% of dogs with previously rapidly progressing disease. These results suggest IDO up-regulation in the tumor micro-environment maintains immunosuppression after immunotherapy. IDO blockade may improve treatment efficacy by limiting this rebound immune-suppression, allowing the local tumor to initiate a systemic anti-tumor immune response. The efficacy and limited toxicity of this strategy are attractive for clinical translation.

